# miRTARGET: An integrated web tool for the identification of microRNA targets with potential therapeutic or prognostic value in cancer

**DOI:** 10.1016/j.neo.2025.101202

**Published:** 2025-06-24

**Authors:** Matjaz Rokavec, Heiko Hermeking

**Affiliations:** a Experimental and Molecular Pathology, Institute of Pathology, Faculty of Medicine, Ludwig-Maximilians-Universität München, Munich, Germany; b German Cancer Consortium (DKTK), Partner site Munich, Germany; c German Cancer Research Center (DKFZ), Heidelberg, Germany

**Keywords:** microRNA, Webserver, Cancer

## Abstract

miRTARGET (https://www.mirtarget.com) is a web tool for the identification of miRNA targets. It integrates experimental miRNA-related datasets and computational algorithms to generate prediction scores for targets of 1744 human miRNAs. The score is based on four dataset categories: mRNA profiling in cells or mice after (1) ectopic miRNA expression or (2) miRNA inactivation by knock-out or knock-down, (3) correlation analyses of mRNA and miRNA expression profiles, and (4) ten computational miRNA target prediction algorithms. Our validation analyses demonstrated a significant enrichment of published/validated miRNA targets among the predicted miRNA targets, underlining the reliability of the miRTARGET prediction score. In addition, miRTARGET integrates cancer-related datasets from primary tumors and cell lines, allowing users to filter/extract miRNA targets based on cancer cell line dependency, survival associations, and differential expression between tumor and normal tissues across 32 cancer entities. As a proof-of-concept, miRTARGET identified *CDC7* and its regulatory unit *DBF4* as the top cancer-associated predicted targets of the tumor suppressive miRNA miR-30a. Therefore, the CDC7-DBF4 complex may represent an attractive candidate therapeutic target for the treatment of cancers with miR-30a inactivation. Altogether, miRTARGET is a powerful and user-friendly web tool for exploring miRNA targets with therapeutic or prognostic potential in cancer.

## Graphical abstract

**Figure:**
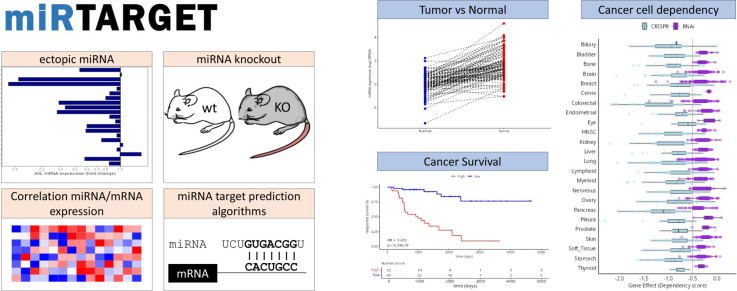

## Introduction

MicroRNAs (miRNAs) are small, non-coding RNA molecules that play a critical role in the post-transcriptional regulation of gene expression, impacting numerous cellular processes such as differentiation, proliferation, apoptosis, and stress responses [1]. They are 20-24 nucleotides in length and exert their regulatory function primarily by binding to complementary sequences in the 3′-untranslated regions (UTRs) of target messenger RNAs (mRNAs), resulting in translational repression or mRNA degradation [2]. It has been estimated that miRNAs regulate the expression of over 60 % of human mRNAs, making them integral to gene regulatory networks [3]. Therefore, the identification of miRNA targets is pivotal for understanding the biological function of miRNAs. Putative microRNA target RNAs are often identified by miRNA prediction algorithms, such as TargetScan [4], based on the presence of sites in 3′-UTRs of mRNAs that match the seed region of miRNAs [5]. Since such an approach is entirely computational it may provide false positive targets. In last years, comprehensive genome-wide miRNA and mRNA expression profiling studies of tumors have been performed, which allow correlation analyzes between miRNA and mRNA expression [6]. These datasets have been integrated with computational algorithms in miRNA portals, such as miRgator, miRnet, and StarBase to improve miRNA target prediction [[7], [8], [9]]. In addition, many genome-wide mRNA profiling studies in cell lines and mouse models after ectopic miRNA expression or miRNA knockout have been performed, allowing further improvement of putative miRNA target identification. However, to our knowledge such datasets have not yet been implemented into miRNA target prediction tools. Therefore, we integrated computational datasets with experimental miRNA-related datasets including studies using ectopic miRNA expression or miRNA knockout to generate prediction scores for targets of 1744 human miRNAs. The dysregulation of miRNA expression has been implicated in a variety of diseases, including cancer, cardiovascular diseases, and neurological disorders. In cancer, for instance, specific miRNAs can function as either oncogenes (oncomiRs) or tumor suppressors [10]. For example, miRNAs of the miR-34 family possess tumor suppressive properties [11,12]. Many miR-34 targets, such as *MET* and *AXL* represent important oncogenes and inhibitors of these proteins have been approved for the treatment of certain types of tumors [13,14]. To identify potential cancer-related miRNA targets, we integrated miRNA-related datasets with cancer-related datasets, such as association with survival, expression in tumor and normal tissue, and cancer cell line dependency. Furthermore, we developed a user-friendly web tool that allows the visualization of datasets and may facilitate the identification of putative miRNA targets with cancer therapeutical or prognostic potential, which is not possible with other miRNA target prediction portals.

## Results and discussion

### Overview of miRTARGET

**Fig. 1:**
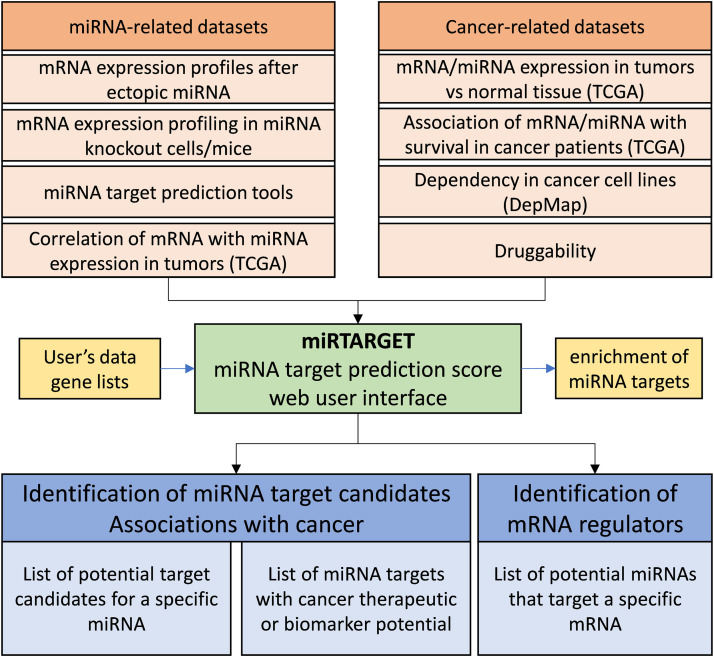
The schematic overview of miRTARGET datasets and workflow.

The schematic overview of miRTARGET is shown in Fig. 1. We included three experimental and one computational miRNA-related dataset categories: 1. 1010 datasets from the NCBI GEO database that represent genome-wide mRNA expression profiles of cell lines and tissues after introduction of ectopic miRNAs by mimics or expression vectors. These datasets correspond to 254 miRNAs with an average of 3.99 studies per miRNA. 2. 334 datasets from the NCBI GEO database that contain genome-wide mRNA expression profiles of cell lines and tissues after miRNA knockout or knockdown. These datasets correspond to 123 miRNAs with an average of 2.73 studies per miRNA. 3. Genome-wide mRNA and miRNA expression profiles from 32 cancer entities (TCGA). 4. Ten computational miRNA target prediction algorithms, such as Targetscan [4]. For datasets from categories 1 and 2 the fold change of each mRNA after ectopic miRNA or miRNA knockdown/knockout was calculated. Since miRNAs generally repress the expression of their targets, mRNAs that are consistently down-regulated by ectopic miRNA and induced by miRNA knockdown/knockout in multiple datasets represent potential miRNA targets. For datasets from category 3, correlation coefficients between the expression of mRNAs and miRNAs were calculated. mRNAs that display a negative correlation with a miRNA represent potential targets of that miRNA. Analyzes of datasets from categories 1 to 3 show whether mRNA expression is correlated/regulated by miRNA. However, these analyzes did not provide information as to whether these regulations are mediated directly via binding of the miRNA to the 3′-UTR of the mRNA. Therefore, we included miRNA target prediction algorithms, which predict miRNA targets by the presence of sites in 3′-UTRs of mRNAs that match the seed region of miRNAs. Next, we integrated datasets from the four categories and calculated a miRNA target probability score (Table S4) and rank (Table S5) for every miRNA/mRNA pair as described in Materials and Methods.

To facilitate the identification of targets with cancer-related therapeutical or prognostic potential, we also included four cancer-related dataset categories: 1. Genome wide profiling of mRNA and miRNA expression in tumors and matching normal tissues from 21 cancer entities (TCGA). The fold change and significance between tumor and normal tissue was calculated for 34334 mRNAs and 1704 miRNAs (Table S6; miRNAs that are significantly up- or down-regulated in tumors compared to normal tissue can also be shown in the miRTARGET website under the tab “CANCER ASSOCIATED miRNAs”). 2. Genome wide association of mRNA and miRNA expression with overall survival in cancer patients from 32 entities (TCGA). The Hazard ratio and significance was calculated for 34334 mRNAs and 1704 miRNAs (Table S7; miRNAs that are significantly associated with overall survival can also be shown in the miRTARGET website under the tab “CANCER ASSOCIATED miRNAs”). 3. Cancer cell line dependency (from the Cancer Dependency Map) for the identification of potential cancer vulnerabilities. 4. Druggability, based on Jiang et al. [15]. The miRNA- and cancer-related datasets have been integrated, which allows miRTARGET users to quickly identify miRNA targets with therapeutic or prognostic potential in cancer, without manual data assembly or processing, saving significant time and effort.

### Benchmarking of miRTARGET miRNA target prediction score

**Fig. 2:**
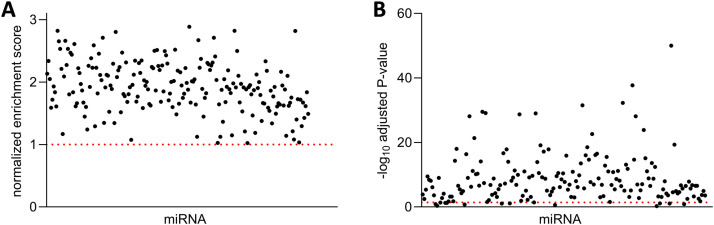
Benchmarking of the miRTARGET miRNA target prediction score. Ranked GSEA Normalized enrichment score (A) and p-value (B) of validated miRNA targets in all mRNAs ranked according to the miRTARGET miRNA prediction score.

Many published, experimentally validated miRNA targets showed high miRTARGET miRNA prediction scores and ranks. For example, the miR-34a target *MET* [16] is ranked as the best miR-34a target and the miR-21 target *PDCD4* [17] is ranked as the second best miR-21 target according to the miRTARGET miRNA prediction score. To systematically test whether miRNA targets with high miRTARGET prediction scores are enriched for validated miRNA targets, we searched the miRTarBase 10.0 [18] for miRNAs with at least 10 targets that were experimentally validated by luciferase 3′-UTR reporter assays, which is a gold standard for miRNA target validation and identified 176 miRNAs that fulfilled this criterion. For each of these miRNAs, we performed a ranked GSEA analysis to test the enrichment of validated targets in all mRNAs ranked according to the miRTARGET miRNA prediction score for that miRNA. For 164 from 176 miRNAs the validated miRNA targets were significantly enriched in mRNAs with high miRNA target prediction scores for that miRNA, suggesting that the miRTARGET score can accurately identify miRNA targets (Fig. 2A and B).

**Fig. 3:**
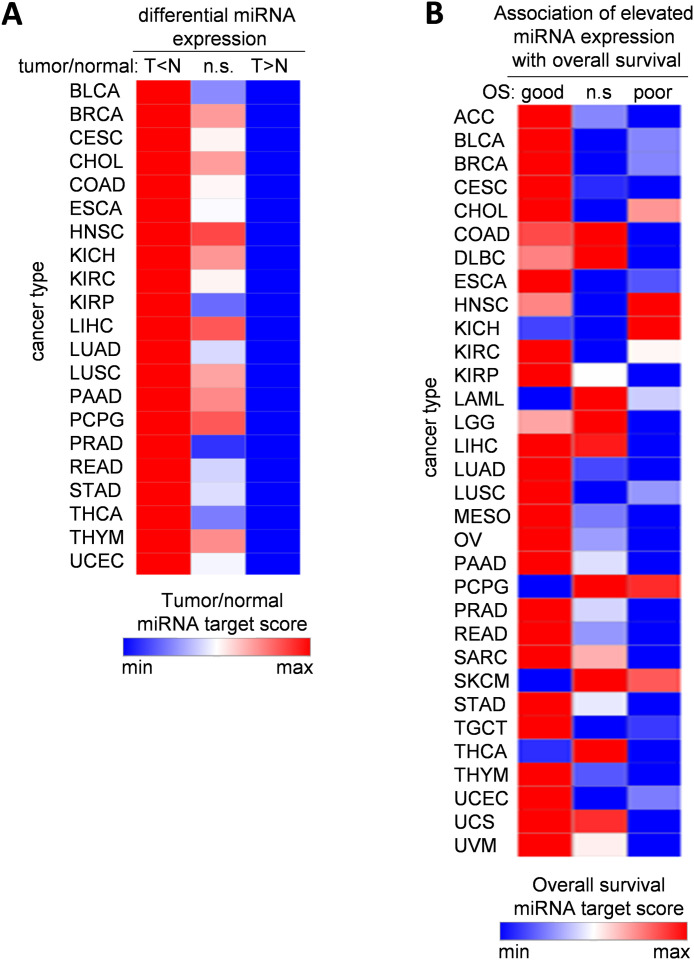
Correlations between clinical associations of miRNAs and their targets. (A) Expression of miRNAs and their targets in tumor versus normal tissues from indicated cancer types. For miRNAs that were downregulated in tumors compared to normal tissue (*T* < *N*), their top1000 predicted targets were predominantly upregulated in tumors (high Tumor/normal miRNA target score), whereas targets of upregulated miRNAs (*T* > *N*) displayed lower expression (low Tumor/normal miRNA target score) in tumors from all analyzed cancer entities. (B) Associations of miRNAs and their targets with overall survival in patients with indicated cancer types. For miRNAs that were associated with good survival (OS: good), their top1000 predicted targets were predominantly associated with poor survival (High Overall survival miRNA target score), whereas targets of miRNAs associated with poor survival (OS: poor) were associated with good survival (low Overall survival miRNA target score) in the majority of cancer entities. For details regarding the calculations of Tumor/normal and Overall survival miRNA target scores see materials and methods (section Correlations between clinical associations of miRNAs and their targets).

Next, we analyzed the correlations between clinical associations of miRNAs and their targets. For miRNAs that were downregulated in tumors compared to normal tissue, their top1000 predicted targets were predominantly upregulated in tumors, whereas targets of upregulated miRNAs displayed lower expression in tumors from all analyzed cancer entities (Fig. 3A). Similarly, the top1000 predicted targets of miRNAs associated with good survival were associated with poor survival, whereas targets of miRNAs associated with poor survival were targeted by miRNAs associated with good survival in the majority of cancer entities (Fig. 3B). These results suggest that targets of tumor suppressive miRNAs have oncogenic properties and vice versa.

### Example applications

#### Identification of miRNA targets with therapeutic applications in cancer

**Fig. 4:**
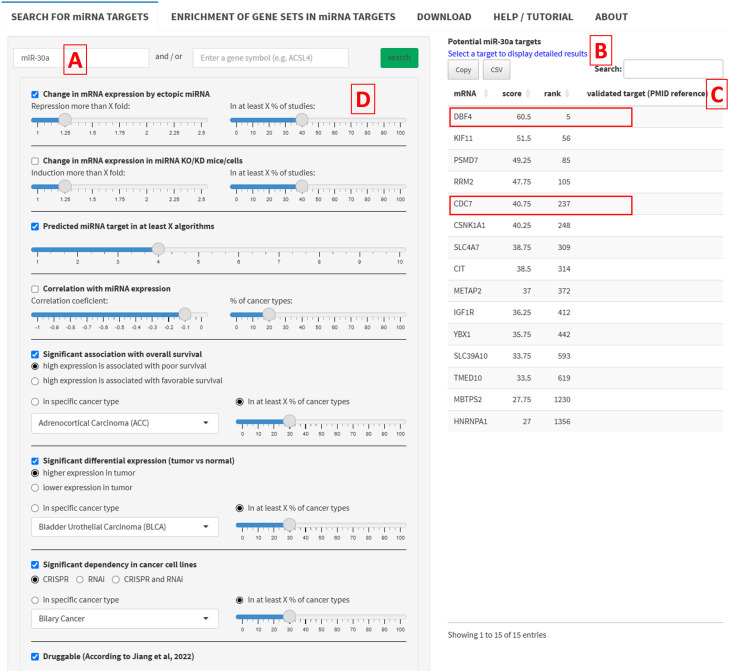
Data input and selection of criteria for miRNA target identification. (A) Input field for miRNA selection (miR-30a). (B) Output of predicted miR-30a targets. (C) Experimentally validated miR-30a targets. (D) Control panel for the selection of miRNA- and Cancer-related criteria.

**Fig. 5:**
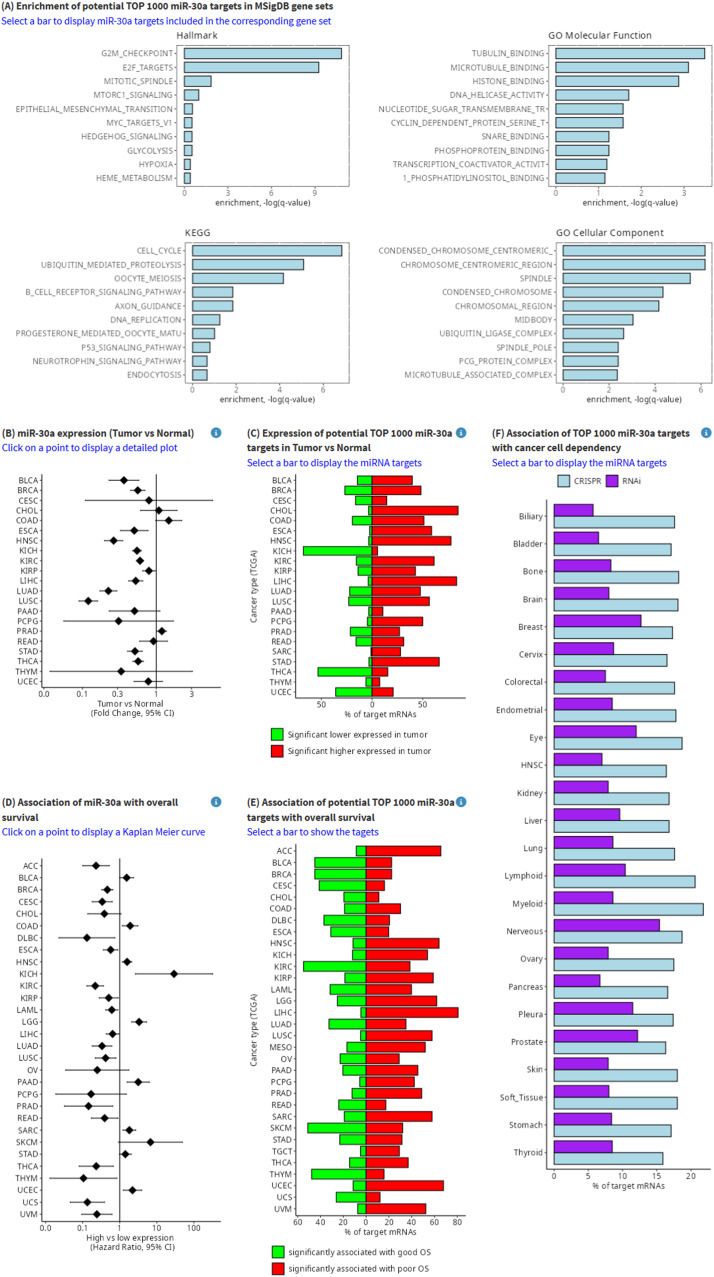
Example application: Identification of miRNA Targets with Therapeutic Applications in Cancer. (A) Enrichment of the top 1000 potential miR-30a targets in indicated MSigDB gene sets. (B) miR-30a expression in tumor versus normal tissue from indicated cancer types. (C) expression of the top 1000 potential miR-30a targets in tumor versus normal tissue from indicated cancer types. (D) Associations of miR-30a expression with overall survival in indicated cancer types. (E) Associations of the top 1000 potential miR-30a targets with overall survival in indicated cancer types. (F) Associations of the top 1000 potential miR-30a targets with cancer cell dependency in indicated cancer types.

Many miRNAs display tumor suppressing properties and are inactivated in tumors [19]. Therefore, therapeutic replacement of miRNAs represents an attractive option for cancer therapeutics. However, it is challenging to target miRNAs in a clinical setting, especially tumor suppressing miRNAs, since they would have to be replaced to achieve tumor suppression [20]. Hence, an alternative approach is the inhibition of up-regulated targets of tumor suppressive miRNAs. One of the possibilities of miRTARGET is the identification of druggable clinically relevant targets of miRNAs. For example, miR-30a acts as a tumor suppressor in many cancer types, including colorectal, breast, renal, and ovarian cancers, where it is frequently downregulated in tumors [21]. To identify potential targets of miR-30a users can enter miR-30a in the search field of the miRTARGET starting page (Fig. 4A). Then, a table is displayed, which shows all miR-30a target mRNAs ranked according to the target prediction score (Fig. 4B). Hence, the most likely potential targets of miR-30a are on the top of the list. The column “validated miRNA target” shows the pubmed ID (PMID) reference if the candidate target has already been experimentally validated according to the miRTarBase 10.0 (Fig. 4C). The plots on the right provide additional information about the predicted miRNA targets (Fig. 5): The top1000 potential miR-30a targets are enriched in G_2_M-checkpoint, E2F targets, and cell cycle related gene-sets (Fig. 5A). The expression of miR-30a is significantly lower in tumor vs normal tissue (Fig. 5B) and associated with good survival in the majority of cancer entities (Fig. 5D). Consistently, the majority of the top1000 predicted miR-30a targets are expressed higher in tumors (Fig. 5C) and associated with poor survival (Fig. 5E). Finally, approximately 15 % and 10 % of miR-30a targets show a dependency in cancer cell lines based on CRISPR and shRNA DepMap screens, respectively (Fig. 5F).

The list of potential miRNA targets can be adjusted by selecting different miRNA- and cancer-related criteria and cutoff values, which are all integrated in one search field allowing users to quickly identify miRNA targets with therapeutic or prognostic potential in cancer, without having to assemble or process the data manually, saving considerable time and effort (Fig. 4D). To identify cancer-associated miR-30a targets users can select “Significant association with overall survival” and/or “Significant differential expression (tumor vs normal)” (Fig. 4D). For oncogenic miRNA targets “high expression is associated with poor survival” and/or “higher expression in tumor” should be selected. To further filter the candidates for potential therapeutic targets, users can select “Significant dependency in cancer cell lines”. Next, users can choose a specific cancer type or select “in at least X % of cancer types” to identify candidates that are associated with multiple cancers. Finally, users can also select “Druggable” to further filter candidates for druggability. Application of these criteria and cutoff values resulted in a list of 15 candidates for druggable clinically relevant targets of miR-30a (Fig. 4B). The top candidate is *DBF4*, which encodes for the regulatory unit of the CDC7 kinase that regulates DNA replication [22]. Interestingly, according to the miRTARGET algorithm, *CDC7* is also a predicted miR-30a target. Therefore, the CDC7-DBF4 complex may represent an attractive candidate therapeutic target for the treatment of cancers with miR-30a inactivation. Indeed, CDC7 has been recognized as a potential target for therapeutic interventions in various cancers and multiple CDC7 inhibitors have been developed [23].

**Fig. 6:**
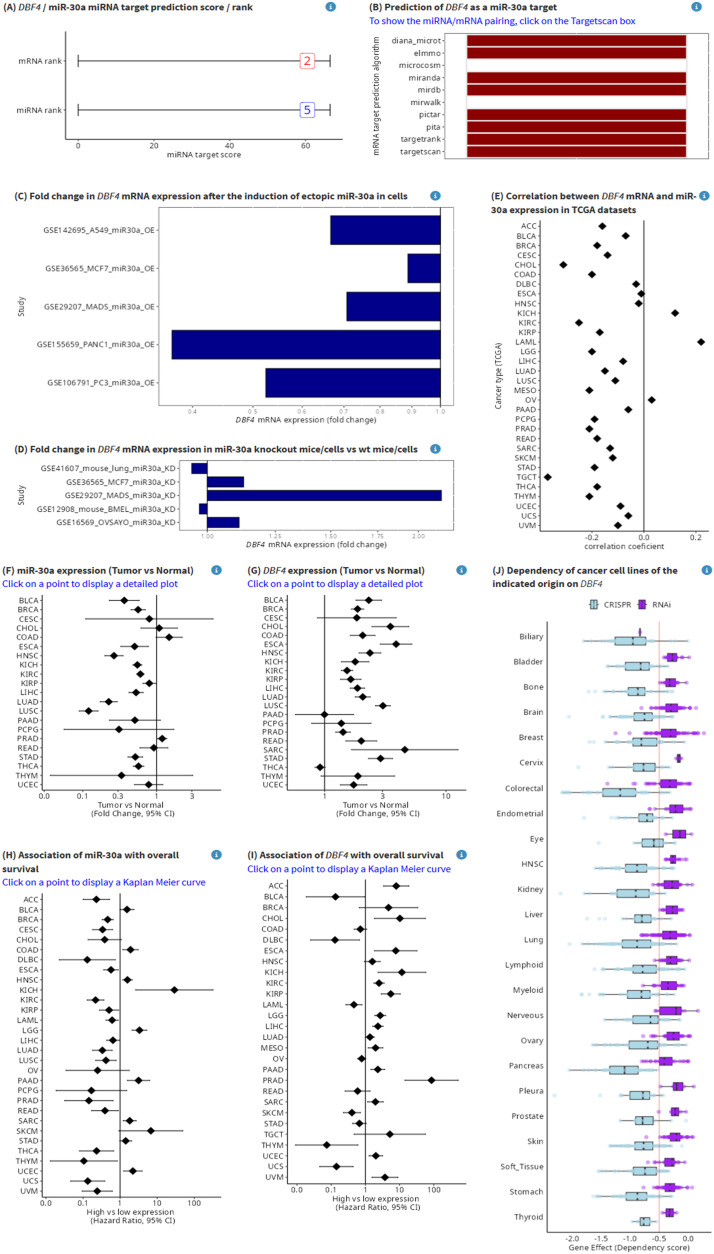
Detailed results for a specific predicted miRNA target. (A) The miRNA and target mRNA score/rank for the miR-30a/*DBF4* miRNA/mRNA pair. (B) Computational prediction of *DBF4* as a miR-30a target based on indicated algorithms. (C) Fold change of *DBF4* mRNA expression by ectopic miR-30a in indicated cells. (D) Fold change of *DBF4* mRNA expression in miR-30a knockout/knockdown cells or tissues in indicated cells. (E) Correlation between miR-30a and *DBF4* expression in tumors from indicated tumor types. Expression of miR-30a (F) and *DBF4* (G) in tumor versus normal tissue from indicated cancer types. Associations of miR-30a (H) and *DBF4* (I) with overall survival in indicated cancer types. (J) Associations of *DBF4* with cancer cell dependency in indicated cancer types.

To obtain detailed results for a specific predicted miRNA target, users can click on the target in the table of miRNA target candidates. miRTARGET will then display multiple plots of miRNA- and cancer-related data in intuitive, straightforward plots that are displayed on one page, allowing to evaluate miRNA- and cancer-related properties of targets at a glance (Fig. 6): (A) The miRNA and the target mRNA score/rank. The miRNA rank for *DBF4* is five, which indicates that *DBF4* is the 5th highest ranked potential target of miR-30a according to the miRTARGET score. The mRNA rank for *DBF4* is two and shows that miR-30a is the 2nd highest ranked potential miRNA regulator of *DBF4*. (B) computational miRNA target prediction, which indicates that eight from ten algorithms predict *DBF4* as a miR-30a target. (C) fold change of target mRNA expression by ectopic miRNA, which shows that *DBF4* is repressed by ectopic miR-30a in five different cancer cell lines / studies. (D) fold change of mRNA expression in miRNA knockout/knockdown, indicating that *DBF4* is induced by miR-30a knockdown in three from five studies. (E) correlation between miRNA and mRNA expression, indicating that the expression of *DBF4* and miR-30a is negatively correlated in 29 from 32 tumor tissues from indicated cancer entities. Furthermore, associations of miR-30a and *DBF4* with cancer properties are shown: (F and G) associations width overall survival in indicated cancer types, indicating that miR-30a is predominantly associated with good survival (F), whereas *DBF4* is associated with poor survival in the majority of cancer types (G); (H and I) differences in expression between tumor and normal tissue in indicated cancer types, indicating that miR-30a is predominantly downregulated in tumors (H), whereas *DBF4* is up-regulated in tumors form the majority of cancer types (I); (J) dependency of cancer cell lines from indicated cancer entities, indicating that cell lines across multiple cancer type are dependent on *DBF4*. The associations with clinical data (F – I) are displayed as clickable forest plots, which provides an overview of associations in all cancer types and detailed presentation of data after clicking on a specific cancer type.

#### Identification of miRNAs that potentially regulate a specific mRNA

**Fig. 7:**
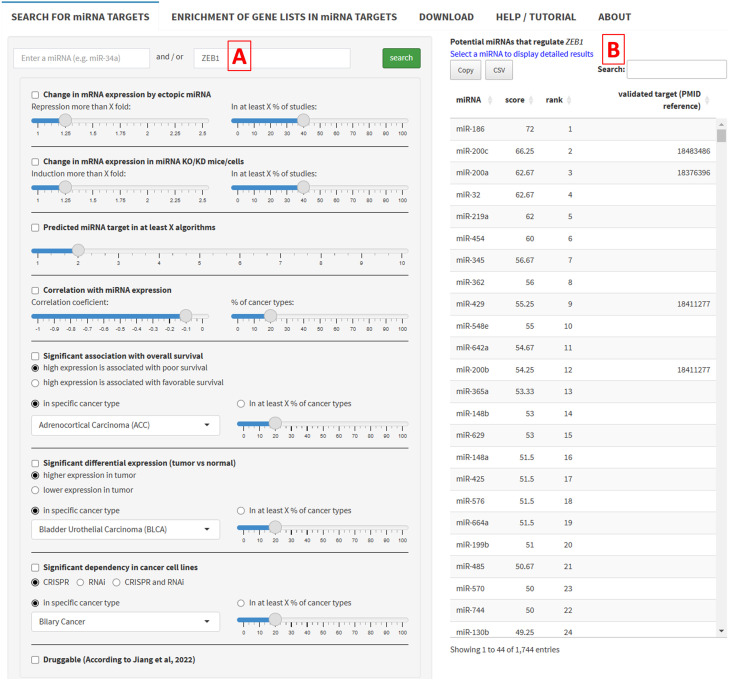
Data input and selection of criteria for the identification of miRNAs that regulate a specific mRNA. (A) Input field for mRNA selection (*ZEB1*). (B) Output of miRNAs that potentially regulate *ZEB1*.

miRTARGET also allows the identification of miRNAs that potentially regulate a specific mRNA (Fig. 7). For this purpose, users can enter a gene/mRNA symbol in the search field (Fig. 7A) (e.g. *ZEB1*). A table (Fig. 7B) shows a list of miRNAs that potentially regulate *ZEB1* ranked according to the target prediction score. The list can be adjusted by selecting criteria and cutoff values as described above.

#### Analysis of user’s data

**Fig. 8:**
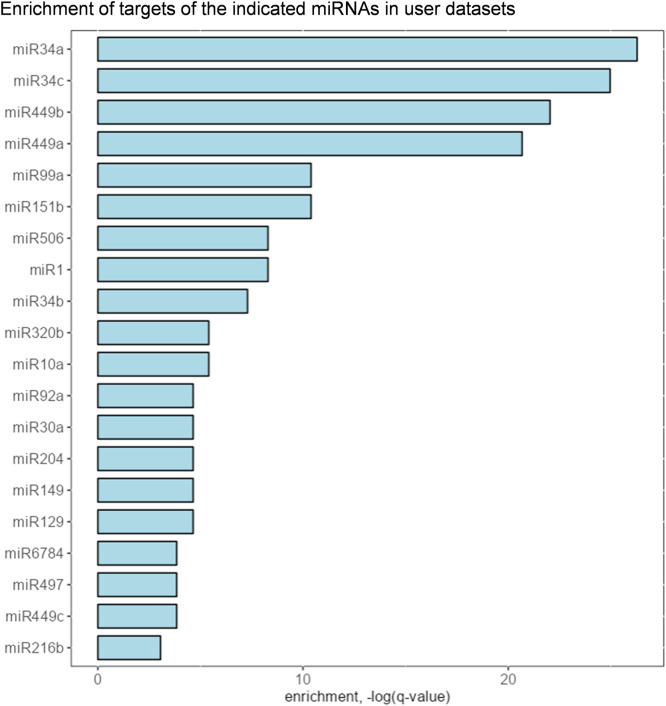
Analysis of user’s data. The enrichment of potential targets of indicated miRNA in 50 most downregulated mRNAs from MDA-231 breast cancer cells transfected with miR-34a mimics.

Another feature of miRTARGET is the analysis of user’s own data. For example, users can upload a list of differentially expressed mRNAs from mRNA profiling experiments and miRTARGET will calculate whether potential targets of any miRNA are enriched in the provided list of mRNAs, thereby providing information on the involvement of specific miRNAs in user’s datasets. As an example, we utilized a list of 50 most downregulated mRNAs from a mRNA profiling study using the MDA-231 breast cancer cells transfected with miR-34a mimics [24]. The most enriched miRNA targets were miR-34a targets and targets of miR-34c, miR-449b, and miR-449a, which all belong to the miR-34 family (Fig. 8).

## Methods

### Data sources and processing

The NCBI GEO (https://www.ncbi.nlm.nih.gov/geo/) database was searched for genome-wide mRNA expression profiling datasets that are based on ectopic miRNA expression or miRNA knockout/knockdown. 1010 datasets obtained from cell lines after transfection with miRNA mimics or expression vectors (Table S1) and 334 datasets derived from miRNA knockout/knockdown cells/mice (Table S2) were identified and downloaded. For datasets using RNA-seq, RPKM values were used and for datasets using microarrays, normalized signal intensity values were used. The fold change was calculated by dividing mRNA expression levels after ectopic miRNA or miRNA knockout/knockdown by the control expression levels for every mRNA. Standardized TCGA datasets containing mRNA and miRNA expression profiling of tumors from 32 cancer entities were downloaded from the MD Anderson MBatch Omic Browser (bioinformatics.mdanderson.org/MQA). The correlation between the expression of each mature miRNA and every mRNA was calculated by Pearson correlation coefficient. Data from 10 miRNA target prediction tools/algorithms was downloaded and analyzed from webpages listed in Table S3. Validated/published miRNA targets were derived from the miRTarBase v10 database [25] (mirtarbase.cuhk.edu.cn/∼miRTarBase/miRTarBase_2025) with the requirement of the validation by luciferase reporter assay experiment.

Clinical data for TCGA datasets from 32 cancer types was obtained from Liu et al. [26]. The statistics for overall survival analysis was calculated by log-rank test. For binary classification of cases (high/low expression), the Survminer R-package (https://CRAN.R-project.org/package=survminer) was used to determine optimal cutoff values. For some miRNAs and mRNAs the cutoff value could not be determined due to low expression. The fold change and significance of the differential expression between tumors and adjacent normal tissue was calculated using limma [27]. For some miRNAs and mRNAs the differential expression could not be determined due to low expression. Expression data from normal tissue was available for 21 cancer types. Cancer cell dependency datasets were retrieved from the Cancer Dependency Map (DepMap; https://depmap.org/portal/). A lower gene effect/dependency score means that a cell line is more dependent on the selected gene. A score of less than −0.5 indicates a strong dependency and a score of less than −1 suggests essentiality. In miRTARGET, a significant dependency on a specific gene is considered if at least 10 % of cell lines of a cancer type exhibit a dependency score of less than −0.5 for that gene. The CRISPR and RNAi indicates whether the data is based on CRISPR (Chronos) or shRNA (DEMETER2) screening. Druggability was assessed based on Jiang et al. [15].

### Calculation of miRNA target prediction score

The algorithm for the calculation of miRNA target prediction scores was adapted from our previous meta-analysis of miR-34 targets, where it was initially described [28]. The miRNA-related datasets (without cancer-related datasets) described above were integrated to calculate a score for every miRNA/mRNA pair in order to estimate the likelihood of a mRNA to represent a potential miRNA target. First, partial scores were calculated for each dataset type. For datasets generated after ectopic miRNA expression the score was calculated as the number of datasets in which the mRNA expression was repressed by >1.25-fold divided by the total number of datasets and multiplied by 100. Many datasets are based on experiments that were performed in unicates (no replicates) and therefore, it was not possible to calculate p-values in order to choose a cutoff based on significance. The fold change of 1.25 was chosen because we consider a decrease of mRNA levels by >25 % as biologically relevant. For miRNA knockout/knockdown datasets the score was calculated as the number of datasets in which the mRNA expression was induced >1.25-fold, divided by the total number of datasets and multiplied by 100. The TCGA mRNA/miRNA correlation score was calculated as the number of datasets/cancer types in which the mRNA/miRNA Pearson correlation coefficient is lower than −0.1 divided by the total number of datasets/cancer types and multiplied by 100. For miRNA target prediction tools/algorithms datasets, the score was calculated by dividing the number of prediction tools predicting a mRNA as a miRNA target by the total number of prediction tools (10) multiplied by 100. The maximum possible partial score for each dataset type is therefore 100. All partial scores were added together with all dataset types weigh equally to obtain the miRNA probability score. The score was divided by 4 in order to designate the maximum possible score to 100. miRTARGET prediction score = ($\frac{no.\;of\;ectopic\;miRNA\;datasets\;(FC<0.8)}{no.\;of\;all\;ecotpic\;miRNA\;datasets}$*100 + $\frac{no.\;of\;miRNA\;knockout\;datasets\;(FC>1.25)}{no.\;of\;all\;miRNA\;knockout\;datasets}$*100 + $\frac{no.\;of\;mRNA/miRNA\;correlation\;datasets\;(r<-0.1)}{no.\;of\;all\;mRNA/miRNA\;correlation\;datasets}$*100 + $\frac{no.\;of\;miRNA\;prediction\;algorithms\;(predicted\;target)\;}{no.\;of\;all\;miRNA\;prediction\;algorithms}$ *100) / 4

For miRNAs for which no datasets with ectopic miRNA or miRNA knockout were available, the score was calculated by adding the partial scores from correlation datasets and datasets from prediction algorithms and divided by two.

### Benchmarking of miRNA target prediction scores

First, we searched the miRTarBase 10.0 [18] for miRNAs with at least 10 targets that were experimentally validated by luciferase 3′-UTR reporter assays. For each of these miRNAs, a ranked GSEA analysis was performed by using the fgsea R package to test the enrichment of validated targets in all genes ranked according to the miRTARGET miRNA prediction score for this miRNA.

### Correlations between clinical associations of miRNAs and their targets

The 1000 top ranked miRNA targets according to the miRTARGET score for every miRNA were extracted. The expression of the top1000 targets for every miRNA was analyzed in tumor and matched normal tissue in TCGA datasets from 21 cancer entities for which expression profiles of normal tissue was available. A score of 1 was designated to targets that were significantly upregulated in tumors and a score of −1 to targets that were significantly downregulated in tumors. For each miRNA a tumor/normal miRNA target score was calculated as a SUM of the scores of all top1000 targets. Hence, a high tumor/normal miRNA target score for a specific miRNA indicates that the top1000 ranked targets of that miRNA are predominantly upregulated tumors, whereas a low tumor/normal miRNA target score indicates that the top1000 targets are predominantly downregulated in tumors. Similar analyzes were performed for associations with overall survival. A score of 1 was designated to targets that were significantly associated with poor survival and a score of −1 to targets that were significantly associated with good survival. For each miRNA an overall survival miRNA target score was calculated as a SUM of the scores of all top1000 targets. Hence, a high overall survival miRNA target score for a specific miRNA indicates that the top1000 ranked targets of that miRNA are predominantly associated with poor survival, whereas a low overall survival miRNA target score indicates that the top1000 targets are predominantly associated with good survival.

### Gene set enrichment analysis

The enrichment of miRNA targets in Human Molecular Signatures Database (MSigDB) collections “Hallmark”, “KEGG”, “GO – Molecular Function”, and “GO – Cellular Component” is calculated by the enricher function from the clusterProfiler R package. The enrichment of gene sets provided by users is calculated by the enricher function from the clusterProfiler R package by using the top1000 predicted targets for each miRNA as the TERM2GENE argument.

### miRTARGET webpage

The miRTARGET webpage was designed using the R package shiny (https://shiny.rstudio.com/).

## Data availability

miRTARGET is freely available at https://www.mirtarget.com and does not require user registration or login.

## CRediT authorship contribution statement

**Matjaz Rokavec:** Writing – review & editing, Writing – original draft, Visualization, Validation, Supervision, Software, Resources, Project administration, Methodology, Investigation, Formal analysis, Data curation, Conceptualization. **Heiko Hermeking:** Writing – review & editing, Writing – original draft, Visualization, Supervision, Project administration, Investigation, Conceptualization.

## Declaration of competing interest

The authors declare that they have no known competing financial interests or personal relationships that could have appeared to influence the work reported in this paper.

## Appendix Supplementary materials

Image, application 1

Image, application 2

Image, application 3

Image, application 4

Image, application 5

Image, application 6

Image, application 7

## References

[1] Krol J., Loedige I., Filipowicz W. (2010). The widespread regulation of microRNA biogenesis, function and decay. Nat. Rev. Genet..

[2] Dong H., Lei J., Ding L., Wen Y., Ju H., Zhang X. (2013). MicroRNA: function, detection, and bioanalysis. Chem. Rev..

[3] Friedman R.C., Farh K.K., Burge C.B., Bartel D.P. (2009). Most mammalian mRNAs are conserved targets of microRNAs. Genome Res..

[4] Agarwal V., Bell G.W., Nam J.W., Bartel D.P. (2015). Predicting effective microRNA target sites in mammalian mRNAs. Elife.

[5] Khatun M.S., Alam M.A., Shoombuatong W., Mollah M.N.H., Kurata H., Hasan M.M. (2022). Recent development of bioinformatics tools for microRNA target prediction. Curr. Med. Chem..

[6] Jacobsen A., Silber J., Harinath G., Huse J.T., Schultz N., Sander C. (2013). Analysis of microRNA-target interactions across diverse cancer types. Nat. Struct. Mol. Biol..

[7] Cho S., Jang I., Jun Y., Yoon S., Ko M., Kwon Y., Choi I., Chang H., Ryu D., Lee B. (2013). MiRGator v3.0: a microRNA portal for deep sequencing, expression profiling and mRNA targeting. Nucleic Acids Res..

[8] Chang L., Zhou G., Soufan O., Xia J. (2020). miRNet 2.0: network-based visual analytics for miRNA functional analysis and systems biology. Nucleic Acids Res..

[9] Li J.H., Liu S., Zhou H., Qu L.H., Yang J.H. (2014). starBase v2.0: decoding miRNA-ceRNA, miRNA-ncRNA and protein-RNA interaction networks from large-scale CLIP-Seq data. Nucleic Acids Res..

[10] Calin G.A., Croce C.M. (2006). MicroRNA signatures in human cancers. Nat. Rev. Cancer.

[11] Rokavec M., Li H., Jiang L., Hermeking H. (2014). The p53/miR-34 axis in development and disease. J. Mol. Cell Biol..

[12] Rokavec M., Oner M.G., Li H., Jackstadt R., Jiang L., Lodygin D., Kaller M., Horst D., Ziegler P.K., Schwitalla S. (2014). IL-6R/STAT3/miR-34a feedback loop promotes EMT-mediated colorectal cancer invasion and metastasis. J. Clin. Invest..

[13] Recondo G., Che J., Janne P.A., Awad M.M. (2020). Targeting MET dysregulation in cancer. Cancer Discov..

[14] Zhu C., Wei Y., Wei X. (2019). AXL receptor tyrosine kinase as a promising anti-cancer approach: functions, molecular mechanisms and clinical applications. Mol. Cancer.

[15] Jiang J., Yuan J., Hu Z., Zhang Y., Zhang T., Xu M., Long M., Fan Y., Tanyi J.L., Montone K.T. (2022). Systematic illumination of druggable genes in cancer genomes. Cell Rep..

[16] He L., He X., Lim L.P., de Stanchina E., Xuan Z., Liang Y., Xue W., Zender L., Magnus J., Ridzon D. (2007). A microRNA component of the p53 tumour suppressor network. Nature.

[17] Liao Y.W., Tsai L.L., Lee Y.H., Hsieh P.L., Yu C.C., Lu M.Y. (2022). miR-21 promotes the fibrotic properties in oral mucosa through targeting PDCD4. J. Dent. Sci..

[18] Huang H.Y., Lin Y.C., Cui S., Huang Y., Tang Y., Xu J., Bao J., Li Y., Wen J., Zuo H. (2022). miRTarBase update 2022: an informative resource for experimentally validated miRNA-target interactions. Nucleic Acids Res..

[19] Di Leva G., Garofalo M., Croce C.M. (2014). MicroRNAs in cancer. Annu. Rev. Pathol..

[20] Reda El Sayed S., Cristante J., Guyon L., Denis J., Chabre O., Cherradi N. (2021). MicroRNA therapeutics in cancer: current advances and challenges. Cancers.

[21] Yang X., Chen Y., Chen L. (2017). The versatile role of microRNA-30a in human cancer. Cell Physiol. Biochem..

[22] Saleh A., Noguchi Y., Aramayo R., Ivanova M.E., Stevens K.M., Montoya A., Sunidhi S., Carranza N.L., Skwark M.J., Speck C. (2022). The structural basis of Cdc7-Dbf4 kinase dependent targeting and phosphorylation of the MCM2-7 double hexamer. Nat. Commun..

[23] Irie T., Sawa M. (2023). CDC7 kinase inhibitors: a survey of recent patent literature (2017-2022). Expert Opin. Ther. Pat..

[24] Mackiewicz M., Huppi K., Pitt J.J., Dorsey T.H., Ambs S., Caplen N.J. (2011). Identification of the receptor tyrosine kinase AXL in breast cancer as a target for the human miR-34a microRNA. Breast Cancer Res. Treat..

[25] Cui S., Yu S., Huang H.Y., Lin Y.C., Huang Y., Zhang B., Xiao J., Zuo H., Wang J., Li Z. (2024). miRTarBase 2025: updates to the collection of experimentally validated microRNA-target interactions. Nucleic Acids Res..

[26] Liu J., Lichtenberg T., Hoadley K.A., Poisson L.M., Lazar A.J., Cherniack A.D., Kovatich A.J., Benz C.C., Levine D.A., Lee A.V. (2018). An integrated TCGA pan-cancer clinical data resource to drive high-quality survival outcome analytics. Cell.

[27] Ritchie M.E., Phipson B., Wu D., Hu Y., Law C.W., Shi W., Smyth G.K. (2015). limma powers differential expression analyses for RNA-sequencing and microarray studies. Nucleic Acids Res..

[28] Rokavec M., Huang Z., Hermeking H. (2023). Meta-analysis of miR-34 target mRNAs using an integrative online application. Comput. Struct. Biotechnol. J..

