# Supplementary material for: miRTARGET: An integrated web tool for the identification of microRNA targets with potential therapeutic or prognostic value in cancer
Source: Neoplasia. 2025 Jun 24;67:101202. doi: 10.1016/j.neo.2025.101202 (PMC12226135; doi:10.1016/j.neo.2025.101202)
Supplement: Supplementary file 3 [file mmc3.docx]

Table S3. Description of miRNA target prediction tools/algorithms.

| **Tool/Algorithm** | **Version** | **Website** |
| --- | --- | --- |
| Targetscan | 8.0 | targetscan.org |
| miRDB | 6.0 | mirdb.org |
| miRWalk | 3 | mirwalk.umm.uni-heidelberg.de |
| miRanda | 2010 | microRNA.org |
| PicTar | 2004 | pictar.mdc-berlin.de |
| PITA | 6 | genie.weizmann.ac.il/pubs/mir07/index.html |
| DIANA-microT-CDS | 5.0 | dianalab.e-ce.uth.gr/html/dianauniverse/index.php?r=microT_CDS |
| Targetrank | 2007 | hollywood.mit.edu/targetrank/ |
| microcosm | 5 | www.ebi.ac.uk/enright-srv/microcosm/htdocs/targets/v5/ |
| Elmmo | 5 | http://www.mirz.unibas.ch/miRNAtargetPredictionBulk.php |
